# Clinical impact of a celiac axis stenosis in patients undergoing hepatobiliary surgery

**DOI:** 10.1007/s00423-023-03003-2

**Published:** 2023-07-16

**Authors:** Pawel A. Bieniek, Shadi Katou, Hermann Kraehling, Max Masthoff, Haluk Morgul, Andreas Pascher, Benjamin Struecker

**Affiliations:** 1 Department of General, Visceral and Transplant Surgery, University Hospital Muenster, Waldeyerstrasse 1, 48149 Münster, Germany; 2 Clinic for Radiology, University Hospital Muenster, Albert-Schweitzer-Campus 1, 48149 Münster, Germany

**Keywords:** Celiac axis, Stenosis, Hepatobiliary surgery, Hepatectomy, Postoperative complication

## Abstract

**Background:**

Celiac axis stenosis (CAS) often is an incidental finding in terms of diagnostic for hepatobiliary surgery since most cases remain asymptomatic. It remains unclear whether CAS is a risk factor for postoperative complications after hepatobiliary surgery. Therefore, the aim of this study was to evaluate the impact of an asymptomatic CAS on the postoperative morbidity and survival of patients undergoing hepatobiliary surgery.

**Methods:**

We retrospectively analyzed CT scans and clinicopathological data of 250 consecutive patients undergoing hepatobiliary surgery between 2011 and 2018 in our tertiary center. We compared the postoperative course between patients with and without an incidental CAS as well as their overall survival.

**Results:**

CAS was caused by atherosclerotic stenosis in 16 (64%) patients, by ligamentous stenosis in 4 (16%) and by combined conditions in 5 cases (20%). Mean age of patients in the CAS group was significantly higher in comparison to patients of the non-CAS group (71.0 vs. 59.1 years, *p* < 0.001). Major hepatectomy was conducted in 40% of the CAS patients and 19.6% of non-CAS patients, respectively (*p* = 0.036). Interestingly, no statistically significant differences in postoperative morbidity (40 vs. 46.2%, *p* = 0.673) or in overall survival between the groups (41.3 vs. 51.9 months, *p* = 0.611) were observed.

**Conclusion:**

Our analysis found no correlation between an asymptomatic celiac axis stenosis and postoperative complications or overall survival after hepatobiliary surgery. Which impact the incidental CAS may have in highly complex cases remains unclear. Further studies are needed to identify patients who benefit from CAS treatment before hepatobiliary surgery.

## Introduction

Celiac axis stenosis (CAS) is defined as an extrinsic (caused by median arcuate ligament) or intrinsic (caused by arteriosclerosis) compression of the celiac trunk. Other rare etiologies can be tumor invasion, fibromuscular dysplasia, inflammatory diseases, arterial dissections or other vascular diseases. [1] The reported prevalence of CAS is ranging from 5 to 10%. [2] Many CAS cases are asymptomatic and therefore CAS often is an incidental finding in cross sectional imaging (i.e. CT and MRI) for preoperative assessment. CAS is mostly graded in mild (< 50%) and substantial (≥ 50%) stenosis. [3].

Hemodynamically relevant CAS can be asymptomatic or cause severe symptoms. [4] However, even asymptomatic CAS may influence formation of collaterals or perfusion of the upper abdominal organs (i.e. liver, pancreas, spleen, gaster) which may become relevant in case of abdominal vasoconstriction (e.g. during abdominal surgery or hemodynamic shock). [5, 6] Consequently, recent reports showed that CAS correlated with a higher risk of postoperative morbidity in patients undergoing pancreatic resections. [7, 8] However, it remains unclear whether asymptomatic CAS may be also a risk factor for hepatobiliary surgery and hence no evidence-based recommendations on the pre- or intraoperative management exist so far. Therefore, the aim of this study was to evaluate the impact of an asymptomatic CAS on postoperative morbidity and survival of patients undergoing hepatobiliary surgery.

## Patients and methods

### Patient inclusion criteria and preoperative assessment

**Fig. 1:**
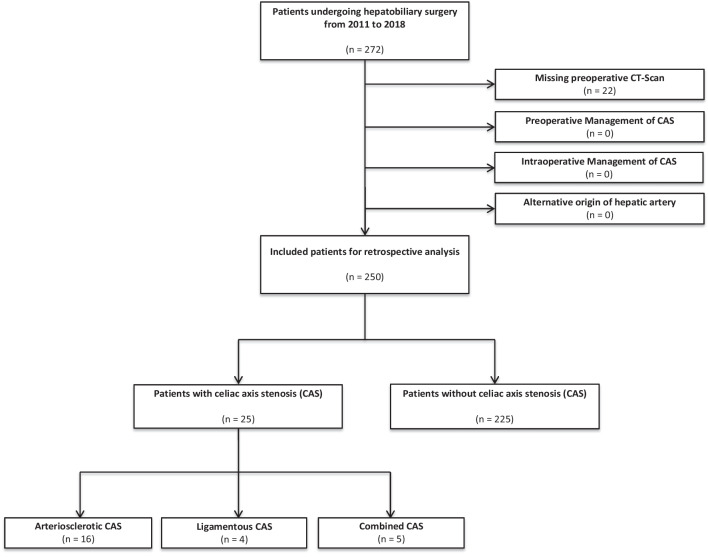
Flow chart of patient selection based on inclusion und exclusion criteria

We retrospectively analyzed data of all consecutive patients who underwent hepatobiliary surgery for different benign and malignant entities between 2011 and 2018 in our tertiary care hepatobiliary surgery center. Patient characteristics were extracted from electronical medical records. The software used to detect and quantify celiac axis stenosis (CAS) was syngo.via (Siemens Healthineers, Erlangen, Germany). Measurement of the CAS was performed according to NASCET (The North American Symptomatic Carotid Endarterectomy Trial) method. [9] CAS was morphologically graded in 1 (< 25%), 2 (25—< 50%), 3 (50—75%) or 4 (> 75%). For clinical relevance classification was carried out in the most common grading system of mild (< 50%) and substantial (≥ 50%) CAS. [3] Patients without preoperative computed tomography were excluded from the study. Furthermore, patients with a preoperative endovascular treatment of an arteriosclerotic CAS or with an intraoperative division of the ligamentum arcuatum also were excluded from the analysis. (Fig. 1) Approval of the local institutional review board was obtained before data were collected and analyzed (Reference Number 2019–636-f-S).

### Surgical procedures

Liver segments were defined according to the classification of Couinaud. [10] Atypical liver resections, segmentectomies and left or right lateral resections were defined as minor hepatectomies. Major hepatectomy was defined as resection of 3 or more segments (i.e. right or left hemihepatectomy, mesohepatectomy and trisectionectomy or two stage hepatectomies according to the portrayal of Couinaud. [11] In all cases vascular resection and lymphadenectomy were performed in a standardized manner.

### Postoperative management

Morbidity was defined as any complication occurring within the hospital stay after hepatobiliary resection and further categorized into surgical and non-surgical complications. Using the Clavien-Dindo classification, major surgical postoperative complications were graded as complications requiring surgical, endoscopic or radiological intervention and life-threatening complications requiring ICU reuptake and intensive care management (grade IIIA or greater). [12] All resected specimens were histologically analyzed by specialized pathologists to evaluate tumor entity and UICC stage. [13, 14].

### Statistical analysis

Quantitative and qualitative variables were expressed as medians (range) and frequencies. Chi-square or Fisher`s exact test and the independent samples t-test were performed to compare categorical and continuous variables, as appropriate. Patients were stratified by the presence of celiac axis stenosis (CAS) and the clinicopathologic characteristics of patients with existing CAS were compared with those of patients without CAS. Overall survival (OS) rates were calculated from the date of hepatobiliary resection to the date of death or last follow-up using Kaplan–Meier method and were compared using Cox proportional hazards model and log-rank test, in univariate analysis.

To identify factors associated with OS in patients with CAS, we evaluated the following clinicopathologic variables in a univariate analysis: sex (male vs. female), patient age at resection (≥ vs. < 70 years), obesity (≥ 30 vs. < 30 body mass index, BMI) [15], alcohol abuse (yes vs. no), diabetes mellitus (yes vs. no), cardiac comorbidity (yes vs. no), pulmonary comorbidity (yes vs. no), renal insufficiency (yes vs. no), liver cirrhosis (yes vs. no), American Society of Anesthesiologists physical status ( I vs. II vs. III vs. IV), operative procedure (minor vs. major resections), operative time (≥ 180 vs. < 180 min), postoperative complications (yes vs. no), posthepatectomy liver failure (yes vs. no), reoperation (yes vs. no), reuptake ICU (yes vs. no) and duration of hospital stay (≥ 14 vs. < 14 days). All variables associated with OS with *p* < 0.05 in the univariate proportional hazard models were entered into a Cox multivariate regression model. *P* < 0.05 was considered statistically significant. Statistical analyses were performed using the software IBM SPSS statistics version 27 (IBM, Armonk, N.Y., USA).

## Results

### Patient characteristics

**Fig. 2:**
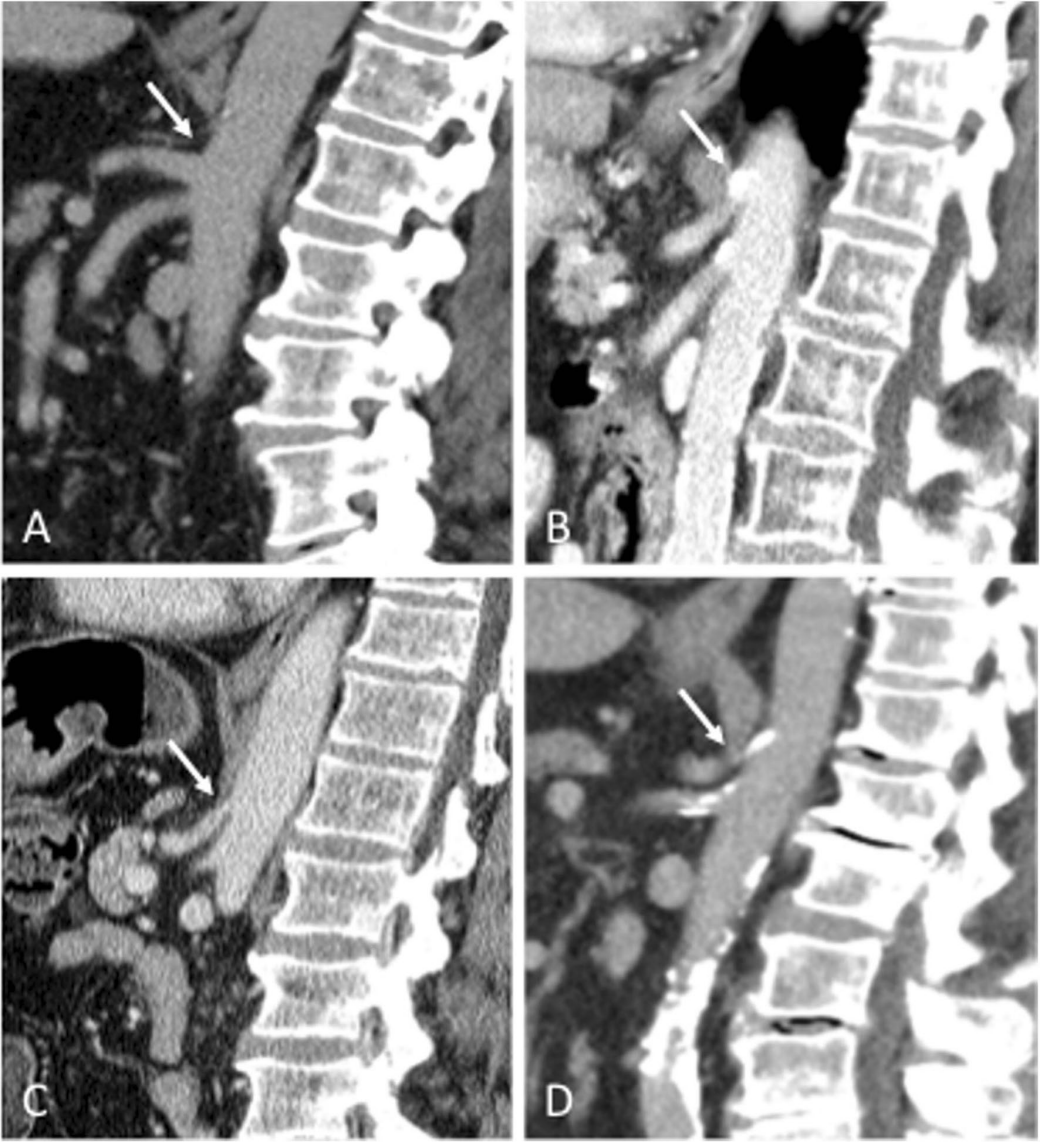
Types of substantial celiac axis stenosis. Preoperative sagittal computed tomography (CT) scan. (**A**) No Celiac axis stenosis (CAS). (**B**) Arteriosclerotic CAS. (**C**) Ligamentous CAS. (**D**) Combined arteriosclerotic and ligamentous CAS

During the study period, 250 consecutive patients who underwent hepatobiliary surgery in our tertiary care center were included in this study. Overall twenty-five patients (10%) were diagnosed with a celiac axis stenosis (CAS) in the preoperative computed tomography (CT) scan. CAS was caused by atherosclerotic stenosis in 16 (64%) patients, by ligamentous stenosis in 4 (16%) and by combined conditions in 5 cases (20%). (Fig. 2) CAS under 50% was defined as mild stenosis, greater than or equal to 50% as substantial. As shown in Table 1, mean percentage stenosis at time of resection was 42.8% (± 18.1%) and mean length of the stenosis was 4.6 mm (± 1.5 mm).

**Table 1:** Characteristics of the celiac axis stenosis

Characteristics	Number (Percentage)
*Celiac axis stenosis*	25 (10%)
*Type of celiac axis stenosis*	
* - Atherosclerotic*	16 (64%)
* - Ligamentous*	4 (16%)
* - Combined*	5 (20%)
*Grade of the celiac axis stenosis*	
* - Grade I (1–24%)*	4 (16%)
* - Grade II (25–49%)*	10 (40%)
* - Grade III (50–74%)*	11 (44%)
* -Grade IV (75–100%)*	0 (0.0%)
*Degree of celiac axis stenosis (%)*	42.8 (7–66) ± 18.1
*Length of celiac axis stenosis in mm*	4.6 (2–7) ± 1.5

Regarding clinicopathologic characteristics as presented in Table 2, the age of patients with and without CAS did significantly differ: The mean age in patients with CAS was 71.0 years whereas the mean age in patients without CAS was 59.1 years (*p* < 0.001). Also, the prevalence of diabetes mellitus (40.0 vs. 19.1%, *p* = 0.021) and cardiac morbidity (52.0 vs. 19.6%, *p* = 0.001) was significantly higher in patients with CAS. The remaining clinicopathologic characteristics showed no statistically significant differences: Sixty percent of patients with CAS and 55.1% of patients without CAS were male, respectively (*p* = 0.641). Median BMI did not significantly differ between both groups (27.3 vs. 26.4 kg/m^2^, *p* = 0.386). The distribution in ASA groups in patients with and without CAS was not significantly different (*p* = 0.295). Although no existing alcohol abuse was documented in the CAS group, there was no significant difference between both groups (0.0 vs. 7.1%, *p* = 0.386). Even though frequency of smoking was greater in cases without CAS, the statistical relevance was not significant (8 vs. 16.9%, *p* = 0.390). A higher fraction of patients with CAS (60.0%) was diagnosed with arterial hypertension in comparison to 42.7% of patients without CAS but differences were not statistically significant (*p* = 0.136). A liver cirrhosis was preexisting in 16% of patients with CAS compared to 8% of patients without CAS (*p* = 0.252). At time of hepatobiliary resection, 8% in the CAS group were suffering from renal insufficiency, in comparison to 6.7% of the CAS group (*p* = 0.682). Pulmonary comorbidity was present in 16% of patients with CAS and in 10.2% of patients without CAS (*p* = 0.326).

**Table 2:** Perioperative data of patients with celiac axis stenosis

Variable	Total n (%)	With Celiac Axis Stenosis n (%)	Without Celiac Axis Stenosis n (%)	p-value
*Male*	139 (55.6%)	15 (60.0%)	124 (55.1%)	.641^†^
*Age year*	60.3 ± 14.5	71.0 ± 12.5	59.1 ± 14.2	<.001^§^
*BMI kg/m*^*2*^	26.5 ± 4.5	27.3 ± 4.3	26.4 ± 4.5	.386^§^
*ASA*				.295^†^
* I*	17 (6.8%)	2 (8.0%)	15 (6.7%)	
* II*	135 (54.0%)	11 (44.0%)	124 (55.1%)	
* III*	67 (26.8%)	9 (36.0%)	58 (25.8%)	
* IV*	2 (0.8%)	1 (4.0%)	1 (0.4%)	
*Habit*				
* Smoke*	40 (16.0%)	2 (8.0%)	38 (16.9%)	.390^‡^
* Alcohol*	16 (6.4%)	0 (0.0%)	16 (7.1%)	.386^‡^
* Comorbidity*				
* DM*	53 (21.2%)	10 (40.0%)	43 (19.1%)	.021^‡^
* ATH*	111 (44.4%)	15 (60.0%)	96 (42.7%)	.136^‡^
* Cardiac*	57 (22.8%)	13 (52.0%)	44 (19.6%)	.001^‡^
* Pulmonary*	27 (10.8%)	4 (16.0%)	23 (10.2%)	.326^‡^
* Renal insufficiency*	17 (6.8%)	2 (8.0%)	15 (6.7%)	.682^‡^
* Infectious diseases*	4 (1.6%)	0 (0.0%)	4 (1.8%)	1.000^‡^
* Liver cirrhosis*	22 (8.8%)	4 (16.0%)	18 (8.0%)	.252^‡^
* Other liver disease*	54 (21.6%)	5 (20.0%)	49 (21.8%)	1.000^‡^
* Other malignancy*	134 (53.6%)	11 (44.0%)	123 (54,7%)	.399^‡^
* Other comorbidity*	145 (58.0%)	19 (76.0%)	126 (56.0%)	0.58^‡^
*Intraoperative data*				
* Operative time min*	225.6 ± 94.8	209.7 ± 58.4	227.5 ± 98.1	.193^§^
* Operative proc*				.036^‡^
* Minor resection*	196 (78.4%)	15 (60.0%)	181 (80.4%)	
* Major resection*	54 (21.6%)	10 (40.0%)	44 (19.6%)	
* Operation type*				.609^‡^
* Laparotomy*	239 (95.6%)	25 (100%)	214 (95.1%)	
* Laparoscopy*	11 (4.4%)	0 (0.0%)	11 (4.9%)	
* Bile duct reconstruction*	12 (4.8%)	2 (8.0%)	10 (4.4%)	.346^‡^
*Pathology*				.063^†^
* HCC*	47 (19.4%)	9 (36.0%)	38 (16.9%)	
* CCC, Klatskin*	37 (15.3%)	7 (28.0%)	30 (13.3%)	
* CRLM*	63 (26.0%)	5 (20.0%)	58 (25.8%)	
* NET*	8 (3.3%)	0 (0.0%)	8 (3,6%)	
* Other malignancy*	41 (16.9%)	1 (4.0%)	40 (17.8%)	
* Benign*	41 (16.9%)	3 (12.0%)	38 (16.9%)	
* Others*	5 (2.1%)	0 (0.0%)	5 (2.2%)	

^*†*^*Chi-squared test, *^*‡*^*Fisher exact test, *^*§*^*t-test*

### Intraoperative characteristics

Major resections were significantly more frequent in the CAS group (40 vs. 19.6%), and in reverse minor resections were significantly less frequent in this group (60 vs. 80.4%, *p* = 0.036). The median duration of operation between cases with CAS and without CAS did not significantly differ (209.7 vs. 227.5 min, *p* = 0.193). All of 25 cases of CAS were operated via laparotomy whereas 214 of 225 (95.1%) cases without CAS underwent laparotomies (*p* = 0.609). Biliary reconstructions were conducted in 8.0% of patients with CAS and in 4.4% of patients without CAS (*p* = 0.346).

### Tumor entity

Patients with and without CAS showed no significant different characteristics for histopathologic tumor entity (*p* = 0.063). Colorectal liver metastases represented the largest proportion of tumor entities at 26.0%, followed by hepatocellular carcinoma at 19.4% and by cholangiocellular carcinoma at 15.3%. [13, 14].

### Postoperative morbidity and mortality

The rate of overall complications did not significantly differ between both groups (40.0 vs. 46.2%, *p* = 0.673). (Table 3) A trend toward more cases of posthepatectomy liver failure (PHLF), defined as an increased international normalized ratio (INR) and concomitant hyperbilirubinemia on or after postoperative day 5, was seen in patients with CAS (12.0%) compared to patients without CAS (2.7%, *p* = 0.50). [16] Postoperative biliary leak was more frequent in CAS patients (12.0%) in comparison to non- CAS patients (6.7%), although the difference was not statistically significant (*p* = 0.403). Postoperative gastroparesis appeared in 9.8% of patients without CAS, whereas no gastroparesis was documented in the CAS group (*p* = 0.142). Patients with and without CAS showed no significantly different rates of surgical complications like abscess formations (0.0 vs. 1.8%, *p* = 1.00), wound infections (4.0 vs. 6.7%, *p* = 1.00) and bleedings (4.0 vs. 3.1%, *p* = 0.575).

**Table 3:** Postoperative outcome of patients with celiac axis stenosis

Variables	Total *n* (%)	With Celiac Axis Stenosis *n* (%)	Without Celiac Axis Stenosis *n* (%)	*p*-value
*Overall complications*	114 (45.6%)	10 (40.0%)	104 (46.2%)	.673^‡^
* PHLF (ISGLS)*	9 (3.6%)	3 (12.0%)	6 (2.7%)	0.50^‡^
* Biliary leak/fistula*	18 (7.2%)	3 (12.0%)	15 (6.7%)	.403^‡^
* Gastroparesis*	22 (8.8%)	0 (0.0%)	22 (9.8%)	.142^‡^
* Abscess*	4 (1.6%)	0 (0.0%)	4 (1.8%)	1.000^‡^
* Wound infection*	16 (6.4%)	1 (4.0%)	15 (6.7%)	1.000^‡^
* Bleeding*	8 (3.2%)	1 (4.0%)	7 (3.1%)	.575^‡^
* Clavien-Dindo IIIA or greater*	46 (18.4%)	4 (16.0%)	42 (18.7%)	.719^‡^
* Non surgical complic*	35 (14.0%)	4 (16.0%)	31 (13.8%)	.762^‡^
* Pneumonia*	4 (1.6%)	2 (8.0%)	2 (0.9%)	.051^‡^
* Bacteremia*	2 (0.8%)	0 (0.0%)	2 (0.9%)	1.000^‡^
Pulmonary embolism	0 (0.0%)	0 (0.0%)	0 (0.0%)	-
* STEMI/NSTEMI*	0 (0.0%)	0 (0.0%)	0 (0.0%)	-
* Urinary tract infection*	15 (6.0%)	1 (4.0%)	14 (6.2%)	1.000^‡^
* ICU stay, days*	4.2 ± 6.1	4.5 ± 6.6	4.2 ± 6.0	.825^§^
* Hospital stay, days*	17.7 ± 12.5	18.6 ± 10.7	17.6 ± 12.7	.685^§^
*Postoperative laboratory values*				
* Delta hemoglobin g/dl*	-3.6 ± 1.9	-4.1 ± 2.0	-3.6 ± 1.9	.212^§^
* Delta leucocytes Tsd./ul*	1.39 ± 4.32	0.95 ± 2.91	1.44 ± 4.46	.587^§^
* Delta creatinine mg/dl*	-0.1 ± 0.5	-0.3 ± 1.2	-0.1 ± 0.4	.340^§^
* Delta thrombocytes tsd./ul*	-64.1 ± 63.0	-67.0 ± 50.3	-63.7 ± 64.4	.807^§^
* Delta GOT (ASAT U/l)*	55.3 ± 226.7	24.2 ± 49.6	59.5 ± 240.6	.495^§^
* Delta GPT (ALAT U/l)*	111.1 ± 163.4	84.1 ± 171.6	114.6 ± 162.6	.433^§^

^*‡*^*Fisher exact test, *^*§*^*t-test*

The postoperative management of major surgical complications including the requirement of reoperation or the need of readmission to an intensive care unit classified as Clavien-Dindo grade IIIA or greater were more frequent in patients without CAS (grade IIIA or greater: 18,7%) in comparison to patients with CAS (grade IIIA or greater: 16,0%) without statistical significance (*p* = 0.719).

Overall non-surgical complications appeared in 16.0% of patients with CAS and 13.8% of patients without CAS (*p* = 0.762). A higher fraction of CAS patients (8.0%) was postoperatively diagnosed with pneumonia, although differences were not statistically significant (*p* = 0.051).

No cases of pulmonary embolism and myocardial infarction were documented in this study group. Urinary tract infection occurred in 4.0% of CAS patients and 6.2% of non-CAS patients (*p* = 1.00).

The median duration of hospital stay (18.6 vs. 17.6 days, *p* = 0.685) as well as the median duration of ICU stay (4.5 vs. 4.2 days, *p* = 0.825) were comparable in both groups. With regards to postoperative laboratory values the incidence of hemorrhage, infection, renal insufficiency and hepatocellular damage as listed in Table 3 did not significantly differ.

### Long-term survival

**Fig. 3:**
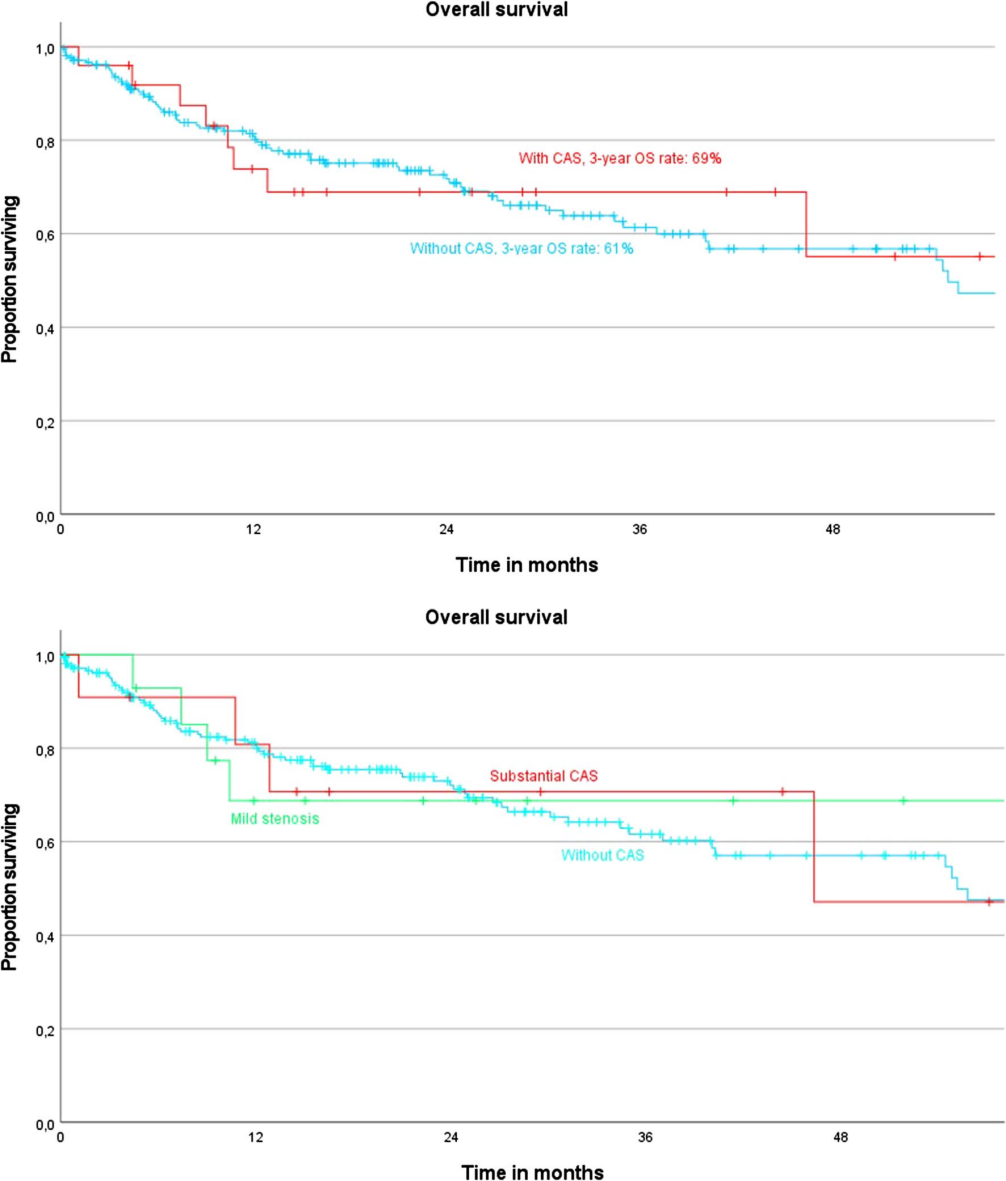
Kaplan Meier curves for comparison of overall survival according to CAS severity

After a mean follow-up time of 22.4 months, the median overall survival for the cohort with CAS was 41.3 months and for the cohort without CAS 51.9 months (*p* = 0.611). The chance of an occurring death for the cohort with CAS was not significantly higher than for the cohort without CAS in our observation period (HR 1.19, *p* = 0.612). To further clarify the impact of the severity of celiac axis stenosis on long-term survival, we analyzed overall survival after grading the CAS in mild and substantial stenosis. Patients with a mild stenosis had a median overall survival of 42.9 months (*p* = 0.766), comparable to that of patients with a substantial stenosis, who had a median overall survival of 41.0 months (*p* = 0.667, Table 4). Hazard ratio for on occurring death for the cohort with a mild CAS (HR 1.56, *p* = 0.756) and also with a substantial CAS (HR 1.24, *p* = 0.644) did not significantly differ with the cohort without CAS in the observation period. The 1- and 3-year OS rates for patients with CAS and without CAS were 69 vs. 78% and 69 vs. 61% (*p* = 0.198 and *p* = 0.935, Table 4, Fig. 3).

**Table 4:** Comparison of overall survival according to CAS severity

Celiac axis stenosis severity	overall survival in months (95% CI)	p-value	hazard ratio (95% CI)	*p*-value
*No stenosis*	51.9 (45.5 – 58.4)			
* Stenosis (Grade I—IV)*	41.3 (31.1 – 51.5)	.611^¶^	1.19 (0.61 – 2.31)	.612
* Mild stenosis (Grade I* + *II)*	42.9 (28.4 – 57.5)	.766^¶^	1.56 (0.47 – 2.87)	.756
* Substantial stenosis (Grade III* + *IV)*	41.0 (25.7 – 56.3)	.667^¶^	1.24 (0.50 – 3.08)	.644

^*¶*^*Log rank test* *Cox proportional hazards model*

### Predictors of overall survival

Detailed analysis of predictors of overall survival in patients with CAS is presented in Table 5. Univariate analysis identified the occurrence of postoperative complications as an independent factor associated with worse 3-year OS (hazard ratio 5.18; 95% confidence interval 1.04 – 25.74; *p* = 0.044).

**Table 5:** Univariate und multivariate analyses of clinicopathologic variables associated with OS in 25 patients with celiac axis stenosis who underwent hepatobiliary resection

*Variable*	*Percent*	*3-Year* *OS, (%)*	*Univariate analysis,* *p value*	Multivariate analysis* p value hazard ratio *(95% CI)*
Gender			.122	
Male	60	79		
Female	40	52		
Patient age at resection, years			.860	
≥ 70	56	70		
< 70	44	68		
BMI, kg/m^2^			.136	
≥ 30	28	100		
< 30	72	57		
Smoking			.392	
Yes	8	50		
No	92	71		
Alcohol abuse			-	
Yes	0	-		
No	100	69		
Diabetes mellitus			.818	
Yes	40	70		
No	60	67		
Cardiac comorbidity			.827	
Yes	52	68		
No	48	71		
Pulmonary comorbidity			.701	
Yes	16	75		
No	84	67		
Renal insufficiency			.406	
Yes	8	-		
No	92	70		
Liver cirrhosis			.322	
Yes	16	75		
No	84	67		
ASA physical status			.808	
I	9	100		
II	48	70		
III	39	53		
IV	4	100		
Operative procedure			.284	
Minor resection	60	60		
Major resection	40	80		
Operative time, min			.160	
≥ 180	72	55		
< 180	28	100		
Postoperative complication			.044	.044 5.18 (1.04–25.74)
Yes	40	50		
No	60	83		
Reoperation			.684	
Yes	4	-		
No	96	67		
Reuptake ICU				
Yes	0	-	-	
No	100	69		
PHLF (ISGLS)			.557	
Yes	12	33		
No	88	74		
Hospital stay, days			.180	
≥ 14	60	53		
< 14	40	100		

^*^Cox regression multivariate analysis included all variables with p < *0.05 in univariate analysis. BMI* = *body mass index; ASA* = *American Society of Anasthesiologists; PHLF* = *posthepatectomy liver failure; ISGLS* = *International Study Group of Liver Surgery; UICC* = *Union international contre le cancer; CI* = *confidence interval. Bold values describe statistically differences between the analyzed groups*

## Discussion

To the best of our knowledge, this is the first study analyzing the impact of CAS on the postoperative outcome after hepatobiliary surgery, since the currently available literature is mainly restricted to case reports or case series regarding patients undergoing pancreaticoduodenectomy. [7, 8, 17-20].

The aim of this study was to evaluate potential correlations between incidence and grade of CAS and postoperative complications with potential ischemic cause (e.g. PHLF, biliary complications etc.). A systematic review conducted by Giovanardi et al. in 2018 showed that approximately 50% of postoperative ischemic complications after pancreatic resections (e.g. ischemic liver damage, pancreatic fistula and biliary leakage) were associated with preexisting CAS. [20] Zhou and Wand et al. analyzed data on patients undergoing pancreaticoduodenectomy and identified a substantial celiac axis stenosis as an independent risk factor for biliary fistula but without an impact on the postoperative course. [7] This finding could be explained by a postoperative reduced perfusion of the common bile duct and consecutively an insufficiency of the hepatico-jejunostomy. By ligation of the gastroduodenal artery and resection of the pancreatic head a potential retrograde blood flow from the superior mesenteric artery to the branches of the celiac axis (via the “Bühler anastomosis”) is impeded. [21] Thus, in cases with relevant CAS a sufficient perfusion of the liver, spleen and gaster maybe hampered after resection of the pancreatic head.

Data from Al-Saeedi et al. supported the increased presence of biliary fistula after pancreaticoduodenectomy by preexisting CAS. [8] In their study postoperative complications as clinically relevant pancreatic fistula, liver perfusion failure, gastric ischemia and prolonged intensive care unit or hospital stay were associated with CAS. Furthermore, Lubrano et al. showed an elevated risk of hepatic artery thrombosis in CAS patients undergoing orthotopic liver transplantation with a greater risk of graft loss, [22] most likely due to a reduced blood flow at the anastomosis.

In our study, 250 patients undergoing hepatobiliary surgery, including 25 patients with CAS, were investigated. Interestingly, the analysis showed that CAS was not significantly associated with postoperative complications in particular with PHLF and biliary leakage and overall survival. This notion may be best explained by maintained retrograde blood flow via gastroduodenal artery fed by collaterals from superior mesenteric artery supplying the upper abdominal organs in case of substantial CAS and hepatectomy, even in case of perioperative abdominal vasoconstriction during and after hepatobiliary surgery.

We identified individual patients in our tertiary center who benefited from a postoperative endovascular dilatation or stenting of an arteriosclerotic CAS after highly complex liver surgery, e.g. ALPPS (associating liver partition and portal vein ligation for staged hepatectomy) or trisectionectomy.

Czigany et al. recommended the intraoperative surgical division of the ligamentum arcuatum in patients with substantial ligamentous CAS because the procedure is feasible and safe and might help to prevent postoperative complications. [23] Our finding were not in accordance with their conclusion. However, so far a consensus on preoperative treatment of an arteriosclerotic CAS is not available for patients undergoing hepatobiliary surgery. A preoperative endovascular stenting of the arteriosclerotic CAS, requires therapeutic anticoagulation, increasing the risk for relevant postoperative bleeding after (major) hepatobiliary resections. [24, 25] Our data does not suggest the need for a preoperative stenting of CAS prior to hepatobiliary surgery. However, patient number of arteriosclerotic CAS is low in our cohort and thus results should be interpreted with caution. Therefore, indication and patient selection for preoperative endovascular stenting of CAS prior to hepatobiliary surgery should be subject to future studies.

In our cohort, patients with CAS were significantly older and suffered more often from coexisting diseases (e.g. diabetes mellitus and cardiac disease). Furthermore, significantly more major resections were performed in patients with CAS. However, a higher postoperative morbidity was not observed in this group.

Interestingly, we identified postoperative complications in patients with CAS as an independent risk factor for a worse 3-year overall survival. This is in accordance with Dorcaratto et al. who found a negative impact of postoperative complications on survival and recurrence after resection of colorectal liver metastases in their meta-analysis from 2019. [26] Due to our relatively small cohort size and subgroup analysis this statement should be interpreted with caution.

The retrospective design and the relatively small cohort size are the main limitations of this study and make it impossible to identify potential subgroups of patients (e.g. patients undergoing ALPPS procedures or trisectionectomies with biliary reconstruction etc.) who would benefit form a preoperative endovascular stenting in case of high-grade arteriosclerotic CAS. Another relevant limitation is the lack of patients with a CAS over 75%. One possible explanation may be a preceded treatment in these patients and thus an exclusion in our study.

A further limitation of this study is that CAS was detected and estimated only by diameter stenosis based on routine CT scan and none of the patients with CAS was evaluated by DSA or in- and expiratory CT scans to objectify if the CAS is hemodynamically relevant. Further, all preoperative CT scans were included for analysis despite potential non-availability of a dedicated arterial phase CT scan, which may hamper grading of CAS. However, CAS detection and grading in mild or substantial CAS was also feasible on venous CT scans.

However, we encourage future comparative studies with larger cohorts to investigate this research question using the same study design. Prospective meta-analysis of observational studies could provide level 2a evidence, the highest evidence that can be accomplished in the context.

Nevertheless, in our study CAS had no significant impact on postoperative morbidity and long-term overall survival after hepatobiliary resection. This indicates that preoperative endovascular stenting of an arteriosclerotic CAS (requiring consecutive therapeutic anticoagulation) should be evaluated carefully before hepatobiliary surgery. In case of ligamentous CAS we cannot support the recommendation to dissect intraoperatively the ligamentum arcuatum during hepatic resection since it has no impact on the postoperative course with regard to our findings although it is a safe and quick procedure and might help to prevent postoperative complications, at least in complex cases.

Astonishingly, most cases of CAS were detected retrospectively, highlighting that CAS is underdiagnosed even in specialized centers.

## Conclusion

In this retrospective analysis of 250 consecutive patients undergoing hepatobiliary surgery in a specialized western center, we found no correlation between an asymptomatic celiac axis stenosis and postoperative complications or overall survival.

A consensus on treatment of an asymptomatic ligamentous or arteriosclerotic CAS is not available so far. An endovascular management of incidentally found CAS requiring a therapeutic anticoagulation should be carefully evaluated before hepatobiliary surgery. The impact of a high grade but asymptomatic CAS in highly complex cases (e.g. ALPPS or trisectionectomies with biliary reconstruction) remains unclear. Thus, further studies are needed to identify patients who benefit from ligamentous or arteriosclerotic CAS treatment before or during hepatobiliary surgery.

## Data Availability

The authors confirm that the data supporting the findings of this study are available within the article and its supplementary materials.
